# Health Disparities in Exercise Performance in Patients with Repaired Tetralogy of Fallot

**DOI:** 10.1007/s00246-024-03706-3

**Published:** 2024-11-07

**Authors:** Andrea L. Jones, Rui Xiao, Ariel A. Williamson, Hadiya Benn, Paul Stephens, Shivani M. Bhatt, Laura Mercer-Rosa, Pamela F. Weiss

**Affiliations:** 1 Division of Cardiology, Department of Pediatrics, Children’s Hospital of Philadelphia, 3401 Civic Center Blvd, Suite 8NW, Philadelphia, PA 19104 USA; 2 Department of Biostatistics, Epidemiology, and Informatics, Children’s Hospital of Philadelphia, Philadelphia, PA USA; 3 The Ballmer Institute for Children’s Behavioral Health, University of Oregon, Portland, OR USA; 4 Drexel University College of Medicine, Philadelphia, PA USA; 5 Division of Rheumatology, Department of Pediatrics, Children’s Hospital of Philadelphia, Philadelphia, PA USA

**Keywords:** Tetralogy of Fallot, Congenital heart disease, Pediatric cardiology, Social determinants of health, Cardiopulmonary exercise testing, Child opportunity index

## Abstract

Black patients with tetralogy of Fallot (TOF) have higher mortality than White patients. Health disparities related to other patient-important outcomes, such as exercise performance, have not been studied in this population. We aimed to determine if there are racial disparities related to exercise performance in patients with TOF and to investigate possible mediators of those disparities. We conducted a retrospective single center study of patients aged 8–25 years with repaired TOF who completed maximal cardiorespiratory exercise tests between 2007 and 2020. The primary outcome was percent predicted oxygen consumption at peak exercise. We used linear regression to determine if race was associated with exercise performance. We used mediation analysis to investigate insurance coverage and neighborhood Child Opportunity Index as possible mediators of this relationship. The study cohort included 163 patients with TOF (136 non-Hispanic/Latinx White and 27 non-Hispanic/Latinx Black). In multivariable analysis, Black patients had a lower percent predicted peak oxygen consumption than White patients by 6.71 percentage points (95% CI − 12.71, − 0.70; p = 0.029). Mediation analysis revealed that the indirect effect of race through insurance coverage accounted for 34.1% of the decrease in exercise performance. Child Opportunity Index was not a statistically significant mediator. Black patients with TOF had worse exercise performance than White patients. Differences in insurance coverage accounted for a significant portion of this difference. Exercise performance is an important outcome for patients with TOF, and further investigation is needed to better understand this disparity and develop interventions to address it.

## Introduction

While outcomes for pediatric congenital heart disease have improved over time, there are significant racial and ethnic health disparities in this patient population [1, 2]. Non-Hispanic/Latinx Black/African American (hereafter, ‘Black’) and Asian pediatric congenital heart disease patients have higher mortality rates than non-Hispanic/Latinx White (hereafter, ‘White’) patients by 1.2 and 1.75 times, respectively [3]. Reductions in mortality rates for Black patients are also lower than that for White patients by 0.3% per year over a 19-year period from 1999 to 2017 [4]. Tetralogy of Fallot (TOF) is one of the most common forms of congenital heart disease, affecting over 1000 infants each year in the United States [5]. Fortunately, survival in patients with TOF is excellent, and over 90% of patients survive to adulthood [6]. However, Black and Hispanic patients with TOF have up to two to three times higher mortality rates in infancy and early childhood than White patients [7, 8].

Although associations between race and ethnicity and survival in patients with congenital heart disease are well documented, research on determinants of theses disparities is limited. Instead of reflecting biological or genetic factors, race and ethnicity are social variables that serve as surrogate markers for an array of adverse societal and environmental factors, including historical and ongoing systemic racism and discrimination [9]. Measures of socioeconomic status (SES), including the Child Opportunity Index (COI), partially mediate racial and ethnic associations with outcomes [3]. Disparities in overall healthcare access and quality of health services are also hypothesized to underlie some of these associations [1].

Diminished exercise performance is one of the most debilitating morbidities in patients with surgically repaired TOF. Diminished exercise performance is multifactorial and is known to be influenced by abnormal right ventricular function, abnormal pulmonary vasculature, and chronotropic impairment, among other factors [10–15]. Reduction in exercise performance begins in childhood and continues to decline over time [16]. Diminished exercise performance is associated with reduced quality of life in children with TOF [17, 18], and prolonged hospitalizations [19] and higher mortality [20, 21] in adult patients with TOF. However, the association of race with exercise performance has not been investigated in this patient population. As race is not a biological variable, racial associations with outcomes likely reflect a complicated combination of factors, including possibly partial mediation by family and neighborhood SES metrics. In particular, with regard to exercise, SES may be implicated as it relates to access to healthy food, neighborhood air quality, and availability of neighborhood outdoor spaces for exercise and play [22–24]. The purpose of this study was to determine the association of race with exercise performance in patients with repaired TOF and test whether SES factors mediate this association.

## Methods

### Ethical Approval

This study was approved by the Children’s Hospital of Philadelphia (CHOP) Institutional Review Board and was deemed exempt from the need to obtain informed consent.

### Study Cohort

We performed a single-center retrospective cohort study of patients who presented to the CHOP exercise testing laboratory for cardiopulmonary exercise testing (CPET) between January 2007 and December 2020. Patients were eligible for inclusion if they fulfilled the following criteria: (1) history of complete surgical repair of TOF, (2) age 8–25 years at the time of CPET, and (3) completion of a maximal CPET, defined as respiratory exchange ratio equal to or greater than 1.1. Subjects up to age 25 were included as patients are routinely referred to our institution’s exercise laboratory from both pediatric cardiology clinics and adult congenital heart disease clinics. Exclusion criteria included patients with absent pulmonary valve or complete TOF repair after age 2 years. Patients with TOF were identified in the electronic medical record using ICD-9 or ICD-10 codes (ICD-9 745.2 and ICD-10 Q21.3). If patients presented for multiple CPET, the first CPET was used. TOF with absent pulmonary valve was excluded as this is a distinct disease process with clinical features dominated by airway compromise rather than right ventricular outflow obstruction. Complete TOF repair was defined as relief of right ventricular outflow tract obstruction and ventricular septal defect closure.

### Clinical Data

Clinical information, including demographics, medical history, surgical history, and echocardiographic data, was manually collected from the CHOP electronic medical record. CPET data was manually collected from the CHOP exercise laboratory database. CPET data included heart rate, blood pressure, oxygen saturation, work rate, and respiratory exchange ratio at peak exercise; oxygen consumption (VO_2_) at anaerobic threshold and peak exercise; and ventilatory efficiency at anaerobic threshold and peak exercise.

### Race and Ethnicity

Race and ethnicity were drawn from the medical record, dichotomized to either non-Hispanic/Latinx Black (‘Black’) or non-Hispanic/Latinx White (‘White’), and considered the primary exposure variable. As noted above, these variables are being used to represent differential exposure to adverse social and environmental factors, rather than as biological indicators. Other racial and ethnic groups were not included due to small sample sizes (n ≤ 13).

### Outcome

The primary outcome was exercise performance, measured as percent predicted peak oxygen consumption (VO_2_). The predicted peak VO_2_ value was based on sex and assumed pubertal status based on patient age [25].

### Socioeconomic Status

**Fig. 1:**
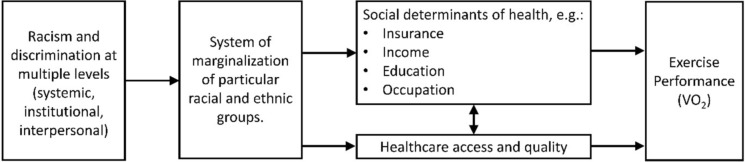
Conceptual model of causal pathways between membership in a marginalized racial group (i.e., Black) and exercise performance. Historical and ongoing structural and institutional racism, in addition to personally mediated racism, contribute to current socioeconomic disparities. Socioeconomic disparities, in turn contribute to health outcomes such as exercise performance through pathways including access to healthcare and differential adverse social and environmental exposures. This is consistent with existing health disparities models, which view social and environmental factors, including differential health coverage, as playing a role in racial health disparities

Measures of SES were considered as potential mediators (Fig. 1) [26]. Race is a social construct, and racism is a root cause of racial disparities that affect health outcomes through a complex array of causal pathways (i.e., mediators), including differential exposure to adverse social and environmental factors [1, 27]. This is consistent with existing health disparities models, which view social and environmental factors, including differential health coverage, as playing a role in racial health disparities [28, 29].

Insurance coverage was used as a proxy for family SES and healthcare access [30]. Insurance coverage was measured at the time of CPET and dichotomized as only private insurance or any public insurance (i.e., public insurance only or both private and public insurance). Medicaid and Children’s Health Insurance Program coverage were considered public insurance [31]. No patients were uninsured. We also reviewed the medical record to determine if patients were in a different insurance category for any prior healthcare encounters.

Neighborhood SES was represented by the Child Opportunity Index (COI), a composite index of 29 indicators of neighborhood SES divided into three domains: education, health and environment, and social and economic [32, 33]. The COI is an established dataset with scores by census tract. It provides a measure of the resources available to children by census tract on a scale of 0 to 100, with 100 representing the highest level of available resources. Geocoding was used to link patient addresses at the time of CPET to census tract to determine the COI.

### Statistical Analysis

All analyses were performed using Stata version 16.1 (StataCorp, College Station, Texas, USA). Descriptive statistics were calculated as frequency counts and percentages for categorical variables and median and interquartile range (IQR) for continuous variables. Distributions of patient characteristics were compared between the two groups by race using Wilcoxon rank-sum test, chi-squared test, and Fisher’s exact test, as appropriate.

Linear regression was used to assess the relationship between race and percent predicted peak VO_2_. Covariates of interest included biological sex, year of birth, pulmonary valve morphology, genetic syndrome, age at complete repair, type of outflow tract repair, history of subsequent pulmonary valve replacement prior to CPET, age at CPET, body mass index (BMI) at CPET, and CPET mode [17, 34, 35]. Biological sex was drawn from the medical record. The final multivariable linear regression model was developed using stepwise forward selection. The final model included variables with a p-value less than 0.05. We also tested the interaction between race and age.

**Fig. 2:**
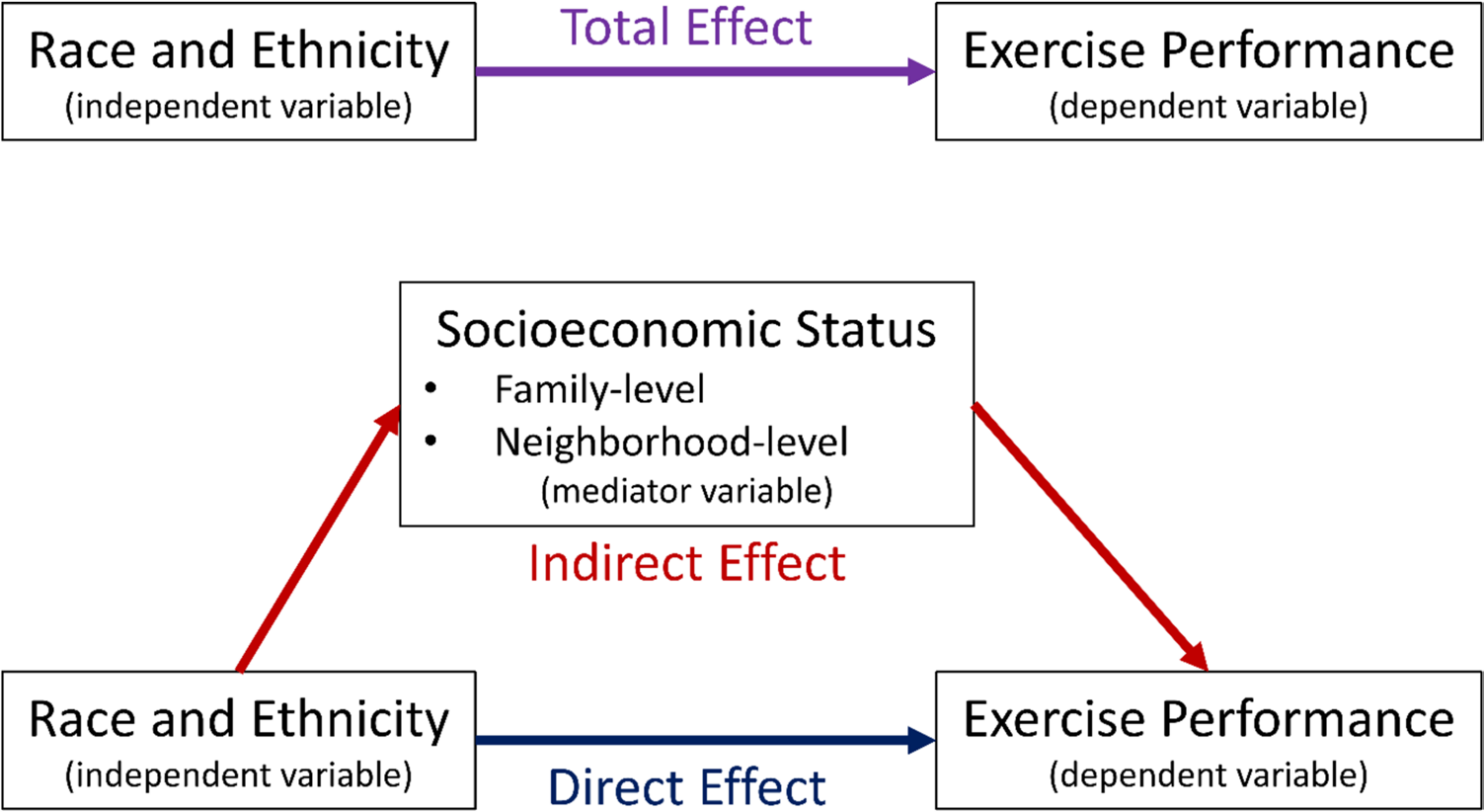
Conceptual model of mediation analysis. Mediation analysis decomposes the total effect of a predictor (independent variable) on the outcome (dependent variable) into the direct effect and an indirect effect through a mediator. We hypothesized that race is associated with exercise performance in patients with repaired TOF and SES mediates this association

Mediation analysis was performed to test the hypothesized mediation pathway of SES indicators (insurance and COI) on the association between race and peak VO_2_ (Fig. 2), in which the total effect of race was parsed into direct and indirect effects. SES indicators were considered as potential mediators rather than confounders as they were hypothesized to be in the causal pathway between race and exercise performance, as described above. For a variable to be considered a statistical mediator, three criteria must be met: (1) there must be a non-null relationship between the exposure variable (e.g., race) and the outcome variable (e.g., peak VO_2_), (2) there must be a significant association between the exposure variable (e.g., race) and the mediator variable (e.g., insurance coverage) and (3) there must be a significant association between the mediator variable (e.g., insurance coverage) and the outcome variable (e.g., peak VO_2_) [36, 37]. First, these component associations were assessed through a series of adjusted linear and/or logistic regressions, as appropriate. Then, effect estimates from models were combined to decompose the total effect of the exposure variable on the outcome variable into its direct and indirect components. The proportion of the total effect attributed to the mediator variable was then calculated.

The 95% confidence intervals (CIs) for the direct and indirect effects were calculated using bootstrapping. The mediator is considered statistically significant if the indirect effect is statistically significant. COI was treated as a continuous variable. Stratified analyses were conducted to determine the association of percent predicted VO_2_ at peak exercise by race within insurance subgroups.

Sensitivity analyses were performed using a maximum age cutoff of 18 years to determine if the relationship between race and exercise performance was robust in an exclusively pediatric cohort and using a minimum respiratory exchange ratio of 1.2 to determine if the relationship was robust with a definition of maximal CPET with higher specificity. Finally, we performed a sensitivity analysis reassigning patients whose insurance coverage category changed within the year prior to CPET.

## Results

### Patient Characteristics

The study cohort included 163 patients with repaired TOF who underwent CPET between January 2007 and December 2020. Patient demographic and clinical characteristics by race are shown in Table 1. Seventeen percent of patients were Black (n = 27), 58.9% were male (n = 96), and the median age at CPET was 13.9 years (IQR: 12.0, 16.6). White patients were more likely to have undergone transannular patch repair (88.0% vs. 70.8%) while Black patients were more likely to have undergone right ventricle to pulmonary artery conduit placement (8.3% vs. 6.0%) or no outflow tract repair or non-transannular patch (20.8% vs. 6.0%; p = 0.037 overall). Black patients were more likely to have undergone pulmonary valve replacement (40.7% vs. 12.5%; p < 0.001). Compared to White patients, Black patients had a lower neighborhood COI (17 vs. 81; p < 0.001) and were more likely to have public insurance (63.0% vs. 23.0%; p < 0.001).

**Table 1:** Patient demographic and clinical characteristics

	Total	Non-Hispanic/Latinx White	Non-Hispanic/Latinx Black	p-value
	N = 163	N = 136	N = 27	
*Clinical Variables*				
Sex				0.966
Male	96 (58.9%)	80 (58.8%)	16 (59.3%)	
Female	67 (41.1%)	56 (41.2%)	11 (40.7%)	
Year of Birth	1997 (1993–2001)	1996 (1993–2001)	2000 (1997–2004)	0.028
Pulmonary Valve Morphology (n = 159)				0.254
Stenosis	133 (83.6%)	108 (81.8%)	25 (92.6%)	
Atresia	26 (16.4%)	24 (18.2%)	2 (7.4%)	
Genetic Syndrome	18 (11.0%)	15 (11.0%)	3 (11.1%)	1.000
Age at Complete Repair (months)	3.5 (1.5–6.9)	3.4 (1.5–6.2)	4.8 (1.7–8.1)	0.265
Outflow Tract Repair (n = 157)				0.037
Transannular Patch	134 (85.4%)	117 (88.0%)	17 (70.8%)	
None or Non-Transannular Patch	13 (8.3%)	8 (6.0%)	5 (20.8%)	
RV-PA Conduit	10 (6.4%)	8 (6.0%)	2 (8.3%)	
Pulmonary Valve Replacement	28 (17.2%)	17 (12.5%)	11 (40.7%)	< 0.001
Age at CPET (years)	13.9 (12.0–16.6)	14.1 (12.4–16.8)	13.1 (9.6–16.6)	0.153
BMI at CPET	19.8 (16.9–23.1)	20.0 (17.2–23.3)	19.5 (15.8–22.0)	0.226
*SES Variables*				
Child Opportunity Index (continuous) (n = 137)	75 (48–89)	81 (65–91)	17 (6–49)	< 0.001
Child Opportunity Index (categorical) (n = 137)				< 0.001
Very Low (1–20)	15 (10.9%)	2 (1.8%)	13 (52.0%)	
Low (21–40)	10 (7.3%)	8 (7.1%)	2 (8.0%)	
Moderate (41–60)	17 (12.4%)	12 (10.7%)	5 (20.0%)	
High (61–80)	36 (26.3%)	33 (29.5%)	3 (12.0%)	
Very High (81–100)	59 (43.1%)	57 (50.9%)	2 (8.0%)	
Insurance Coverage (n = 162)				< 0.001
Any Public Insurance	48 (29.6%)	31 (23.0%)	17 (63.0%)	
Changed insurance category prior to CPET	26 (16.0%)	20 (14.7%)	6 (22.2%)	0.330
Time in insurance category prior to CPET (years)	5.66 (1.57–9.84)	5.13 (1.29–9.85)	7.69 (2.67–9.84)	0.205

Data are presented as median (IQR) for continuous measures, and n (%) for categorical measures; n is presented for variables with missing data. *BMI* body mass index, *CPET* cardiopulmonary exercise test, *PA* pulmonary artery, *RV* right ventricle, *SES* socioeconomic status

Echocardiography and exercise data by race are shown in Table 2. There were no differences in right ventricular function or right ventricular to pulmonary artery coupling, however there was a high degree of missingness for these variables. Black patients had a lower percent predicted heart rate at peak exercise (86.9 vs. 89.7; 0 = 0.042).

**Table 2:** Patient echocardiographic and exercise data

	Total	Non-Hispanic/Latinx White	Non-Hispanic/Latinx Black	p-value
	N = 163	N = 136	N = 27	
*Echocardiographic Variables*				
RV Function (n = 103)				0.654
Normal	81 (78.6%)	64 (80.0%)	17 (73.9%)	
Low Normal	13 (12.6%)	10 (12.5%)	3 (13.0%)	
Mildly Diminished	7 (6.8%)	5 (6.3%)	2 (8.7%)	
Moderately Diminished	2 (1.9%)	1 (1.3%)	1 (4.3%)	
TAPSE/RV pressure estimate^a^ (n = 31)	0.051 (0.036–0.071)	0.055 (0.039–0.076)	0.039 (0.030–0.054)	0.117
*CPET Measurements*				
CPET Mode				0.574
Bicycle	146 (89.6%)	121 (89.0%)	25 (92.6%)	
Treadmill	17 (10.4%)	15 (11.0%)	2 (7.4%)	
Percent predicted VO_2_ at AT	86.8 (73.2–100.7)	88.5 (72.9–102.1)	80.7 (74.2–99.3)	0.510
VE/VCO_2_ at AT (n = 152)	32.5 (29.4–36.3)	32.5 (29.5–36.6)	33.3 (28.8–35.5)	0.726
Respiratory exchange ratio at peak exercise	1.2 (1.2–1.3)	1.2 (1.2–1.3)	1.2 (1.2–1.3)	0.931
VO_2_/kg/min at peak exercise	34.8 (28.5–41.3)	34.3 (29.0–41.0)	35 (25.3–41.1)	0.726
Percent predicted VO_2_ at peak exercise	81.8 (70.0–97.0)	83.3 (71.4–96.8)	76.6 (66.2–100.0)	0.278
VE/VCO_2_ at peak exercise (n = 162)	35.4 (30.9–38.8)	35.4 (31.3–38.8)	35.4 (29.8–39.2)	0.765
Work rate at peak exercise (W/kg) (n = 147)	2.4 (2.0–2.9)	2.4 (2.0–2.9)	2.4 (1.9–2.9)	0.512
Percent predicted heart rate at peak exercise	89.7 (84.7–92.9)	89.7 (85.5–93.3)	86.9 (82.2–90.8)	0.042
Systolic blood pressure at peak exercise (n = 159)	144.0 (132.0–160.0)	144.0 (133.0–156.0)	155.0 (130.0–165.0)	0.260

^a^TAPSE/RV pressure estimates represents a measure of RV-pulmonary artery coupling

Data are presented as median (IQR) for continuous measures, and n (%) for categorical measures; n is presented for variables with missing data. *AT* anaerobic threshold, *CPET* cardiopulmonary exercise test, *RV*, right ventricle, *TAPSE* tricuspid annular plane systolic excursion

Black and White patients with a history of pulmonary valve replacement were further compared. There was no difference in age at pulmonary valve replacement (Black: 6.4 ± 5.8 vs. White: 4.3 ± 4.4 years; p = 0.23), pulmonary valve anatomy (100% pulmonary valve stenosis), age at CPET (Black: 13.8 ± 4.0 vs. White: 15.1 ± 4.2 years; p = 0.45), or prevalence of follow up at our institution (Black: 69.2% vs. White: 83.3%; p = 0.35).

### Percent Predicted VO_2_ at Peak Exercise

The median percent predicted peak VO_2_ for the cohort was reduced (81.8 percent predicted; IQR: 70.0, 97.0). All covariates were considered for the multivariable model with the exception or RV-PA coupling due to a high degree of missingness. The multivariable model included race, sex, pulmonary valve morphology, genetic syndrome, age at CPET, and BMI (Table 3). Year of birth, outflow tract repair type, history of pulmonary valve replacement, and percent predicted heart rate at peak exercise were not significant. In the multivariable model, Black patients had a significantly lower adjusted percent predicted peak VO_2_ with a 6.71 percentage point difference (95% CI − 12.71, − 0.70; p = 0.029) compared to White patients. Each additional year of age was associated with a significantly lower percent predicted peak VO_2_ by 1.37 percentage points (95% CI − 2.11, − 0.62; p < 0.001). Interaction between race and age was not significant (β = 0.71; p = 0.367).

**Table 3:** Univariable and multivariable linear regression models for percent predicted VO_2_ at peak exercise

Variable	Univariable				Multivariable	
	β	95% CI	p	β	95% CI	p
Race and ethnicity			0.429			0.029
Non-Hispanic/Latinx White	ref			ref		
Non-Hispanic/Latinx Black	− 3.23	− 11.29, 4.83		− 6.71	− 12.71, − 0.70	
Female sex	3.80	− 2.27, 9.87	0.218	6.21	1.67, 10.75	0.008
Year of birth	1.29	0.76, 1.83	< 0.001	–	–	–
Pulmonary valve morphology			0.130			0.005
Stenosis	ref			ref		
Atresia	− 6.26	− 14.40, 1.87		− 8.90	− 15.14, − 2.66	
Genetic syndrome	− 19.11	− 28.22, − 10.01	< 0.001	− 18.43	− 25.56, − 11.30	< 0.001
Age at complete repair	− 0.87	− 1.53, − 0.20	0.011	–	–	–
Outflow tract repair			0.055	–	–	–
Transannular patch	ref					
None or Non- Transannular Patch	7.98	− 2.73, 18.69				
RV-PA Conduit	− 11.12	− 23.21, − 0.96				
Pulmonary valve replacement	− 2.82	− 10.77, 5.12	0.484	–	–	–
Age at CPET (years)	− 2.42	− 3.16, − 1.67	< 0.001	− 1.37	− 2.11, − 0.62	< 0.001
BMI	− 2.36	− 2.93, − 1.78	< 0.001	− 1.90	− 2.52, − 1.28	< 0.001
Treadmill CPET	5.68	− 4.10, 15.46	0.264	–	–	–
Percent predicted heart rate at peak exercise	0.43	0.07, 0.79	0.021	–	–	–

Non-Hispanic/Latinx White, pulmonary stenosis, and transannular patch outflow tract repair were chosen as reference groups due to having larger sample sizes. *BMI* body mass index, *CPET* cardiopulmonary exercise test, *PA* pulmonary artery, *RV* right ventricle, *SES* socioeconomic status, *VO*_2_ oxygen consumption

### Mediation Analysis

**Fig. 3:**
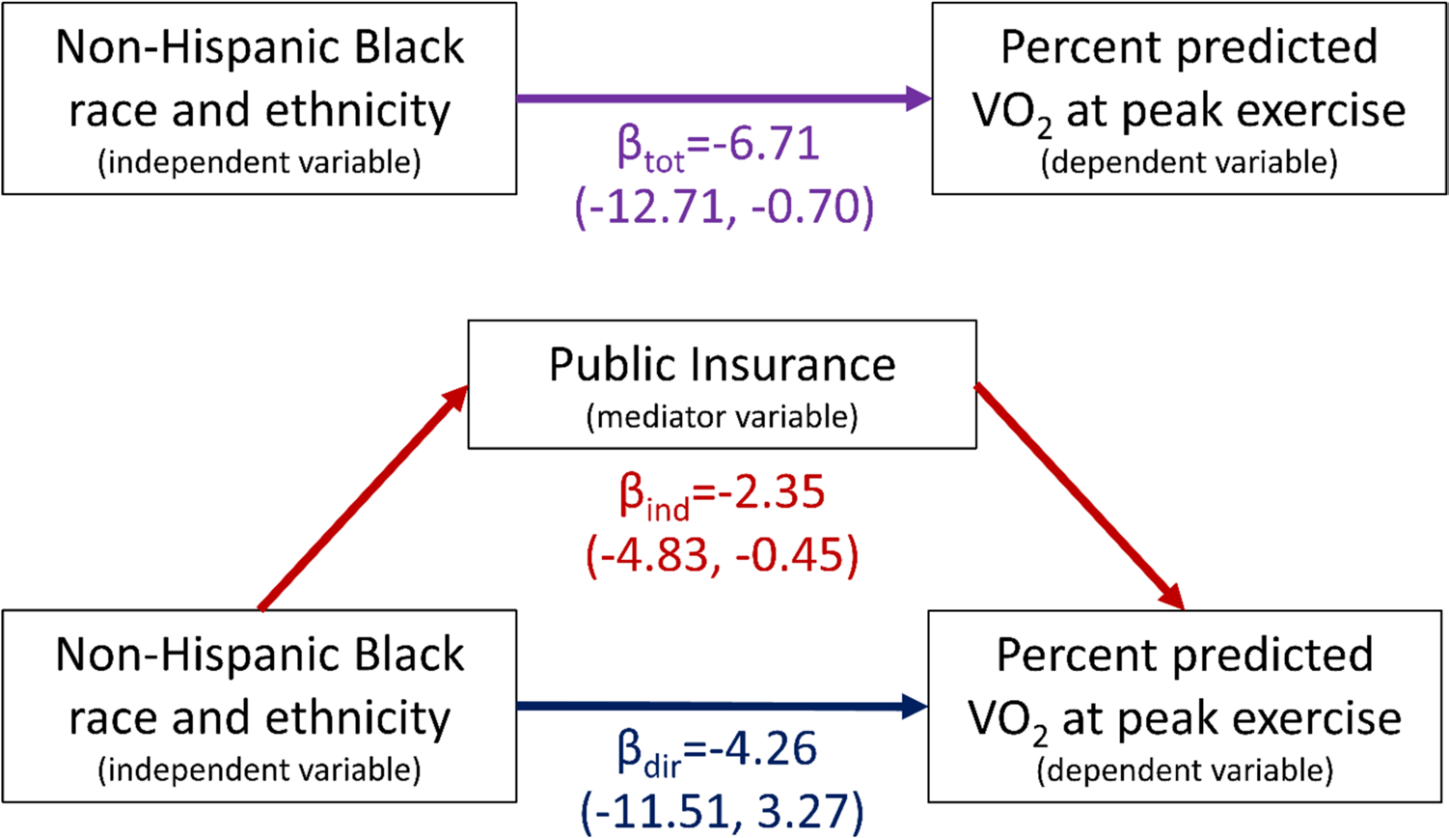
Mediation results. Total, direct, and indirect effect of non-Hispanic/Latinx Black race vs. non-Hispanic/Latinx White race on percent predicted VO_2_ at peak exercise with insurance coverage as a mediator. As a mediator, insurance coverage accounts for 34.1% of the total effect of race on percent predicted peak VO_2_ (p = 0.035)

Mediation analysis was used to decompose the effect of race on percent predicted VO_2_ at peak exercise into direct and indirect components using SES variables as potential mediators. All three criteria were met for insurance coverage to be considered as a mediator. Linear and logistic regressions were adjusted for sex, pulmonary valve morphology, genetic syndrome, age at CPET, and BMI. Mediation analysis showed that insurance coverage mediated 34.1% of the relationship between race and percent predicted peak VO_2_ (Fig. 3; p = 0.035). In this model, the direct relationship between race and percent predicted peak VO_2_ was no longer statistically significant (β = − 4.26; 95% CI − 11.51, 3.27; p = 0.259), while the indirect effect of race on percent predicted peak VO_2_ mediated by insurance coverage was − 2.35 percentage points (95% CI −4.83, − 0.45; p = 0.035).

Stratified analyses were performed within subgroups. White patients with any public insurance had a lower percent predicted peak VO_2_ than White patients with only private insurance by 7.4 percentage points (79.4 ± 17.8 vs. 86.8 ± 19.7; p = 0.073), though this did not reach statistical significance. There was no significant difference in percent predicted VO_2_ at peak exercise for Black patients by insurance (only private insurance, 84.2 ± 19.1 vs. any public insurance, 80.5 ± 19.1; p = 0.353) or within insurance subgroups.

COI as a measure of neighborhood SES did not meet criteria as a mediator, as there was no significant association between COI and percent predicted peak VO_2_ (p = 0.065). No further mediation analysis was performed for this variable.

A sensitivity analysis using an age cutoff of 18 years or younger included 111 White patients and 22 Black patients. Black patients had a lower percent predicted peak VO_2_ by 7.69 percentage points (95% CI − 14.66, − 0.71; p = 0.031). A sensitivity analysis using subjects with a respiratory exchange ratio of 1.2 or greater included 84 White patients and 17 Black patients. In this cohort, the adjusted effect of race on percent predicted peak VO_2_ was similar to the full cohort (β = − 6.07; 95% CI − 13.48, 1.34; p = 0.11). Finally, a sensitivity analysis was conducted by reassigning subjects whose insurance category changed in the prior year (n = 6). The results similarly showed that insurance coverage mediated 36.0% of the total effect (indirect effect β = − 2.46; 95% CI − 5.14, − 0.30; p = 0.046).

## Discussion

In this large single center study, we found that Black patients with TOF had significantly lower exercise performance compared to White patients, and this difference was partly explained by insurance coverage. The mediation analysis approach unveiled a possible mechanism to explain how race is associated with exercise performance, thus providing insight into this observed racial health disparity.

While racial and ethnic disparities among patients with TOF have been documented [1, 2, 7, 38], this study is among the first to specifically examine disparities in exercise performance and to evaluate insurance status and neighborhood disadvantage as potential mediators. Given that race represents differential social and environmental exposures, variation in access to care may be an underlying reason for the observed racial disparity in exercise performance. In this study, public insurance could represent differential access to care, including limited options for specialty care and fewer available resources to support follow up care and adherence to medical recommendations [39–42], which may in turn impact exercise performance. These findings align with prior literature showing that Black children with congenital heart disease had higher emergency care utilization than White children, suggesting limitations to preventative care [43]. Additionally, non-White patients with TOF had higher odds of severe right ventricular dilation on their first cardiac magnetic resonance imaging study, suggesting potential delays in identification and/or referral [38]. Insurance coverage may also be a broader proxy for family-level SES [30, 31], which may impact exercise performance via differential access to healthy foods and options for physical activity [23].

Access to healthy food and options for physical activity may also vary according to neighborhood level SES. Due to historical practices such as redlining and segregation, Black families may be more likely to reside in neighborhoods with fewer resources [44]. In our previous work, we found that neighborhood SES factors (e.g., median household income) were associated with right ventricular function after TOF repair [45]. However, neighborhood level COI, which includes indicators of neighborhood SES, was not a significant mediator for exercise performance, despite being associated with race. This could imply that family-level resources are more important than neighborhood-level resources in determining health-related outcomes. This could also be due to the geographic location of our institution in a relatively accessible area within the city of Philadelphia. These findings may be different at institutions in different environments. Differences across studies regarding neighborhood level factors could also be due to measurement variation, particularly given the multidimensional nature of the COI.

We noted that Black patients had clinically significant differences in exercise performance in both unadjusted and adjusted analyses, however the difference was not statistically significant in the unadjusted analysis. This may have been due to complex relationships between race and the other covariates in the multivariable model. We also noted that Black patients were more likely than White patients to have undergone pulmonary valve replacement prior to CPET, which we were not able to explain based on clinical characteristics. This finding may be indicative of selection bias, as this study was limited to patients who presented for CPET. Additionally, Black patients had a lower percent predicted heart rate at peak exercise, suggesting a higher degree of chronotropic insufficiency. Additional work is needed to better understand this finding.

The American Heart Association recently published a Scientific Statement on Addressing Social Determinants of Health and Mitigating Health Disparities Across the Lifespan in Congenital Heart Disease [1]. Our work is in line with the paradigm shift in which race is viewed as a social rather than biological variable that represents a large and complex web of societal and environmental factors leading to disparate health outcomes in racial and ethnic minoritized individuals. However, it is not enough to identify health disparities. Multi-level interventions must be implemented and evaluated to target modifiable determinants of racial, ethnic, and socioeconomic disparities. Next steps include better delineation of the modifiable factors that contribute to disparities in exercise performance, a clinically important outcome in patients with TOF, to develop programs focused on equitably improving patient outcomes.

## Limitations

Limitations of our study should be acknowledged. First, this was a retrospective study, and we were limited to analysis of variables available in the medical record. Measures of physical activity and more direct measures of family SES, such as household income, parent/guardian educational attainment, and housing type, were not available. In addition, future research should directly measure patient and family experiences of racism and discrimination in medicine rather than utilizing race and ethnicity as proxies for these and other differential social and environmental exposures [46]. Second, this was a cross-sectional study. We collected historical insurance data and attempted to account for a change in insurance status with a sensitivity analysis. However, we did not collect longitudinal CPET results. This limited our ability to evaluate the longitudinal effect of insurance status on exercise performance, particularly for those patients with changes in their insurance coverage. Third, there may be selection bias in that only patients who presented to the exercise laboratory for testing were included in this study. As referrals to the CHOP exercise physiology laboratory are not exclusively from outpatients followed longitudinally in CHOP cardiology practices, we are unable to track patients who did not present for testing. Fourth, this study took place in a large urban academic medical center and our results may not be generalizable to other health care settings. However, our study had similar demographic representation as other studies on patients with congenital heart disease [3]. Fifth, all patients at our institution undergoing CPET have insurance coverage, thus we were unable to evaluate the effect of being uninsured. Finally, despite the setting and the size of the referral base, the number of Black patients included in the study was limited. Sensitivity analyses were performed to assess effect estimates with more restrictive cohort definitions and the effect estimates remained robust. We were also unable to evaluate health disparities in other racial or ethnic groups. Additionally, our ability to detect significant differences within subgroups may have been underpowered due to small sample size. Similar studies may be able to be performed in the future from a larger, possibly multicenter, dataset to address these limitations.

## Conclusion

Racial disparities exist in patients with repaired TOF. We found that Black patients did not perform as well as White patients on CPET after TOF repair and that health insurance coverage partially explained this association. These findings suggest that insurance coverage should be further evaluated in additional research as part of a dedicated effort to elucidate the modifiable factors driving health disparities in exercise performance. This first step is necessary for the future development of targeted interventions to eliminate this disparity.

## Data Availability

The data are available from the authors upon reasonable request.

## Abbreviations

BMI: Body mass index
CHOP: Children’s Hospital of Philadelphia
COI: Child opportunity index
CPET: Cardiopulmonary exercise testing
IQR: Interquartile range
PA: Pulmonary artery
RV: Right ventricle
SES: Socioeconomic status
TOF: Tetralogy of fallot
VO_2_: Oxygen consumption
